# An Efficient Treatment for Rheumatism and Allied Affections

**Published:** 1898-02

**Authors:** C. W. Canan

**Affiliations:** Orkney Springs, Va.


### Therapeutic Notes.

### An Efficient Treatment for Rheumatism and Allied Affections.

BY C. W. CANAN, M. D., ORKNEY SPRINGS, VA.

For the past seven years I have constantly prescribed Tongaline, and the longer I do so the more thoroughly I learn to rely upon its efficacy for the disease for which it is indicated. I had always secured good results from its administration, but during the past year these have far surpassed all my expectations, especially in such serious and obstinate troubles as rheumatism, la grippe and sciatica. These really wonderful results I consider due to my methods of administering the preparation, and I believe it to be to the advantage of every physician to understand just what these methods are. For instance, when I have a very severe case of inflammatory rheumatism, a case where the swelling is great and the pain almost beyond endurance, together with a high temperature, I commence with a teaspoonful of Tongaline every hour in a wineglassful of water just as hot as the patient can bear it. I follow the dose with as much hot water as the patient can take. In from four to eight hours the temperature is invariably reduced and the patient falls into a refreshing sleep. Under this treatment, within six hours I have seen the temperature drop from 104 degrees to 100 degrees and the pain disappear as if by magic. Furthermore, I have time and again witnessed the same results in severe cases of la grippe. The more severe the case, whether of rheumatism, la grippe, gout, sciatica or lumbago, the more I push the Tongaline by giving smaller doses at closer intervals and invariably in hot water in place of cold. In cases where the stomach rebels and Tongaline cannot be administered in that way, I have the affected parts, say the inflamed joints in a case of rheumatism, or the lumbar region in that of lumbago, sponged with alcohol or soda water, in fact, I prefer the latter, then rubbed with Tongaline and apply heat by a hot water bag, or by some other convenient method. It is really surprising how quickly and thoroughly the Tongaline is absorbed and how effective its action when it is administered in that manner. In la grippe, when the stomach is very irritable, as is so often the case, you will find that Tongaline applied locally, say on the inner side of the thighs and under the arms on the side of the chest, will eradicate the trouble more quickly and thoroughly than any other remedial agent.

I call to mind a case of sub-acute, localized rheumatism of the knee, which had defied every kind of treatment generally prescribed for that condition, such as the potassium salts, salicylate of soda, tonics, blisters and counter-irritants. I decided to try Tongaline in the manner above described. By the third day the pain had almost disappeared and the swelling had been reduced two-thirds at least. The improvement was uninterrupted and in ten days the patient pronounced himself cured. It is certainly somewhat remarkable to see an old chronic rheumatic patient, who has been bedridden for months, able to walk comfortably, as if by magic, and due entirely to the effect of Tongaline. On several occasions, when in the company of medical men and the subject of rheumatism was introduced, I have mentioned this treatment, and stated that in my belief we had in Tongaline almost as thorough a specific for rheumatic and neuralgic diseases as quinine was for malaria. Some of the physicians remarked that they had not found Tongaline of so much value, whereupon I replied that the fault was in their manner of prescribing the preparation. I explained to them how Tongaline must invariably be pushed to the extreme in certain obstinate cases and always administered in hot water. Since then I have had the pleasure of hearing one of these physicians state that he is as firm a believer in the efficacy of Tongaline for rheumatism as I am, and that the reason he had never appreciated the preparation so thoroughly was because he had never used it in sufficiently large quantities. In conclusion I would state that if any reader of this article doubts the efficacy that I have ascribed to Tongaline in the more severe forms of the diseases for which it is indicated, just let him push the drug until the full physiological symptoms are secured, and I feel assured that he will agree thoroughly with my statements.—Reprint from the *St. Louis Medical and Surgical Journal.*

